# Supplementary figures and images for: During heat stress in Myxococcus xanthus, the CdbS PilZ domain protein, in concert with two PilZ-DnaK chaperones, perturbs chromosome organization and accelerates cell death
Source: PLoS Genet. 2023 Jun 20;19(6):e1010819. doi: 10.1371/journal.pgen.1010819 (PMC10313047; doi:10.1371/journal.pgen.1010819)

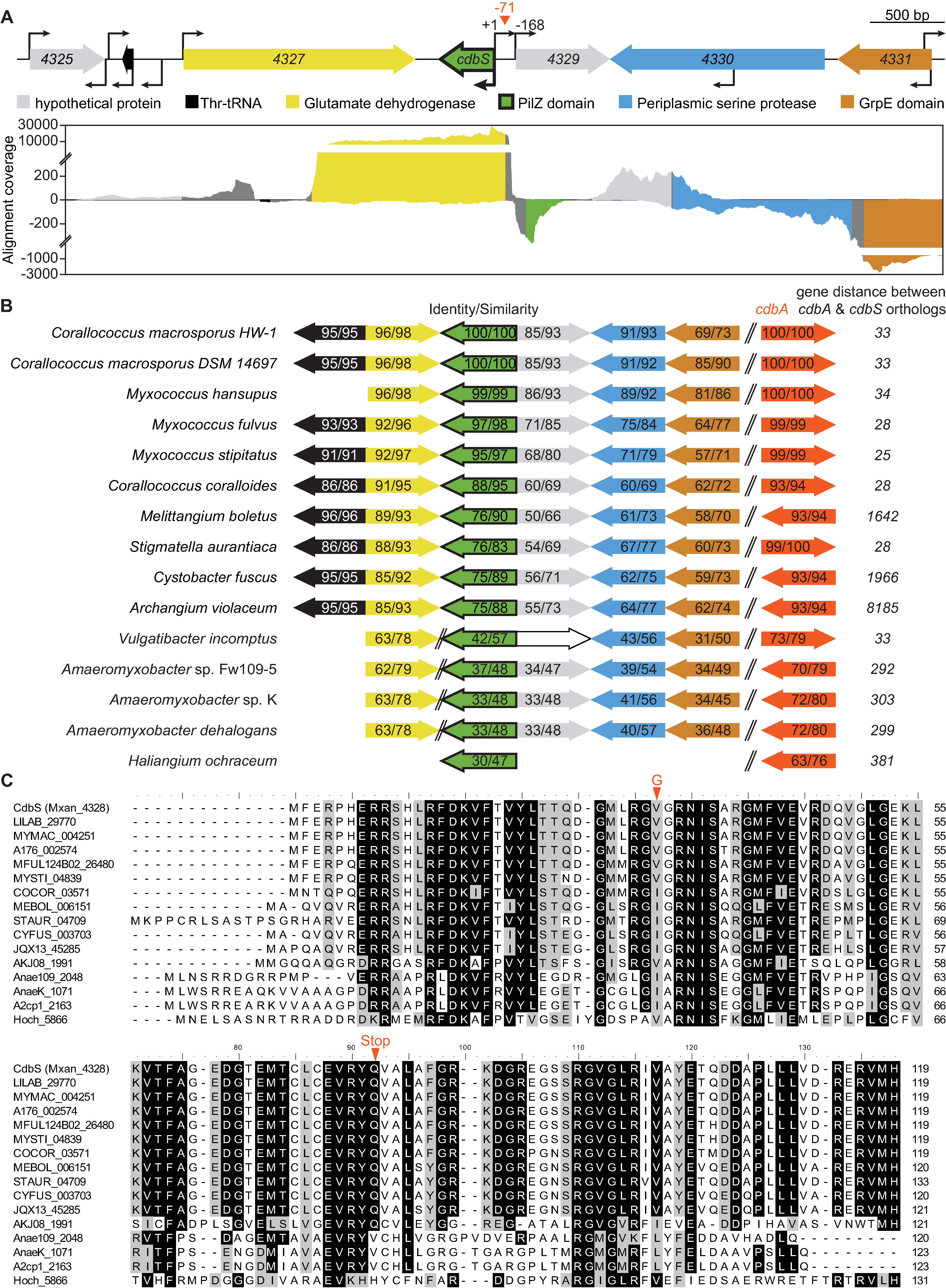

Supplement: S1 Fig — A. cdbS locus. Upper diagram, transcription direction is indicated by the orientation of arrows, kinked arrows indicate transcription start sites as mapped in [24]. Coordinates indicate bp relative to the transcription start site of cdbS. Red triangle indicates the CdbA peak summit from a ChIP-seq analysis in which an active CdbA-FLAG protein was used as bait [11]. The lower diagram show data from RNAseq as base-by-base alignment coverage for total RNA isolated from cells growing in 1% CTT broth [24]. Positive and negative values indicate reads mapped to the forward and reverse strand, respectively. Reads assigned to a gene are colored according to the gene color code in the upper diagram; intergenic regions are in gray. Numbers in genes show mxan_ locus-tags. B. The cdbS locus is conserved in myxobacteria. Transcription direction is indicated by the orientation of arrows with the color used in A. CdbS homologs were identified using reciprocal BLASTP analysis. Numbers indicated % similarity/identity between CdbS of M. xanthus and homologs. % similarity/identity were calculated using EMBOSS Needle software (pairwise sequence alignment). C. Alignment of CdbS proteins. Proteins were aligned with default parameters in MEGA7. Amino acid substitution/stop codon caused by cdbS suppressor mutations are indicated in red. (TIF) [file pgen.1010819.s001.tif]

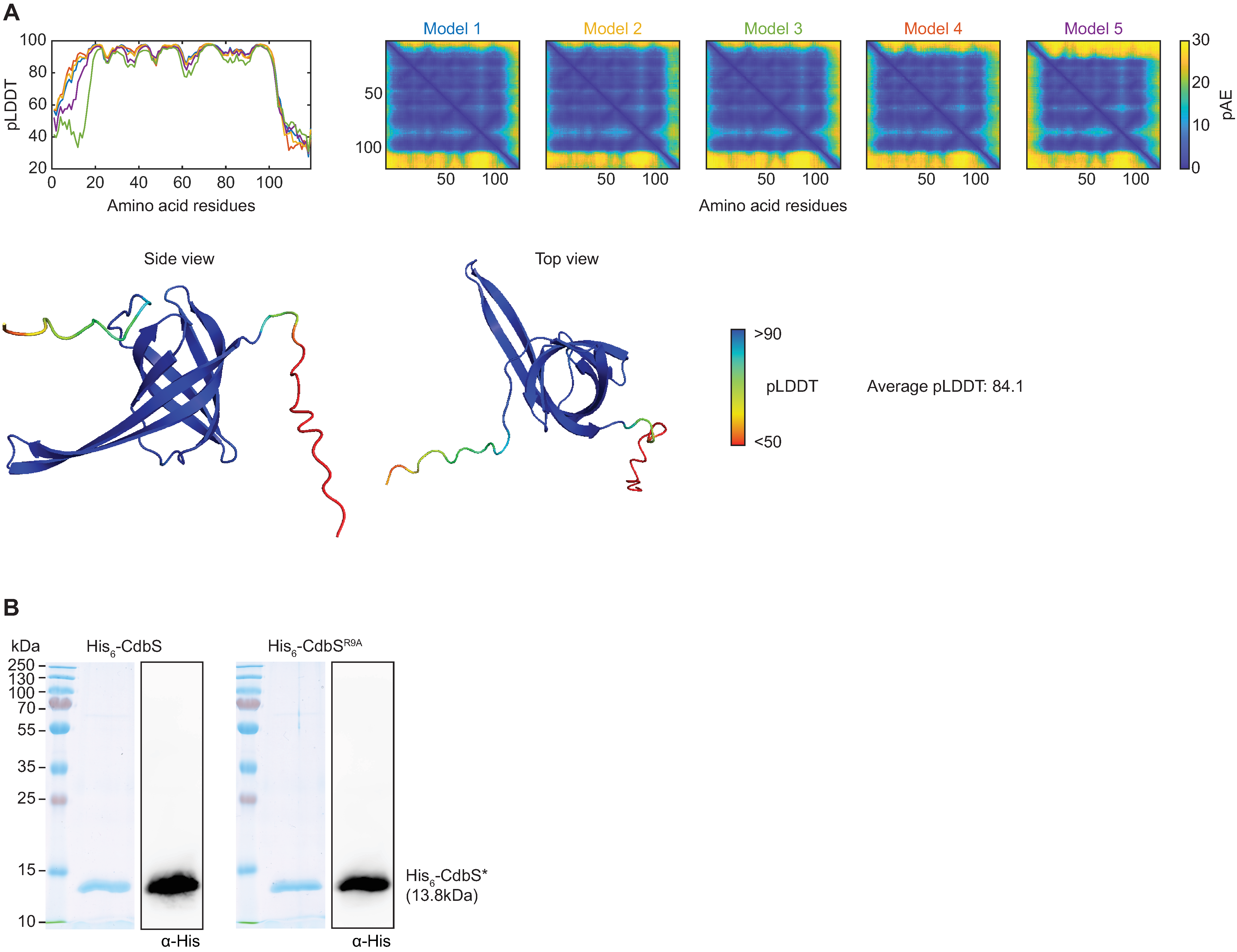

Supplement: S2 Fig — A. pLDDT (Predicted Local Distance Difference Test) and pAE (predicted Alignment Error) plots for five models of CdbS (A) as predicted by AlphaFold. Model rank 1 was used for further analysis and is shown below colored based on pLDDT. B. SDS-PAGE analysis of purified His6-CdbS proteins used in vitro. 1μg of the indicated purified proteins were separated by SDS-PAGE and gels stained with InstantBlue and corresponding immunoblot analysis with α-His6 antibodies. (TIF) [file pgen.1010819.s002.tif]

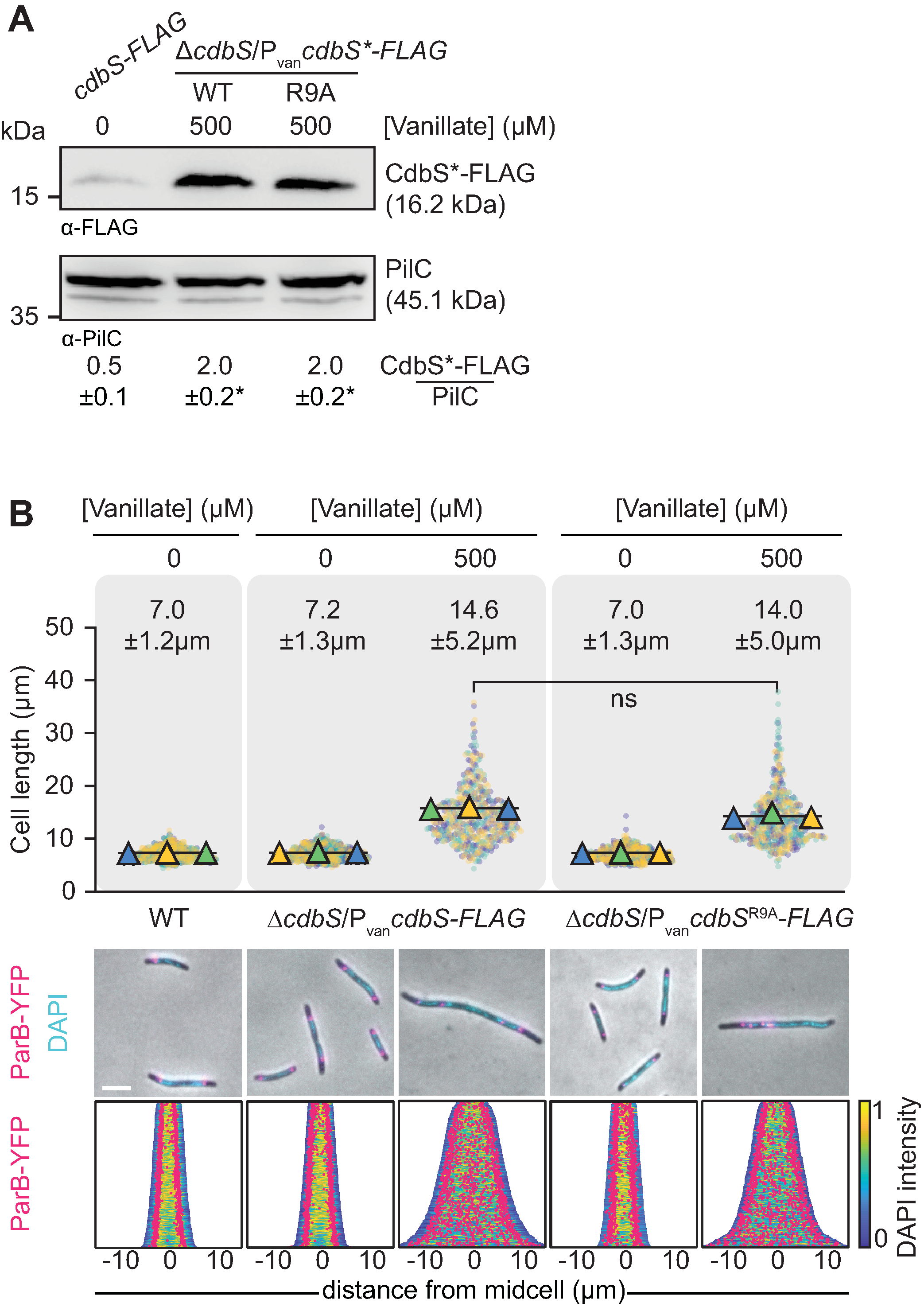

Supplement: S3 Fig — A. Immunoblot analysis of CdbS*-FLAG accumulation. Cells of the indicated genotypes were grown in the presence or absence of the indicated concentrations of vanillate. Cells grown in the presence of vanillate were analyzed 24hrs after addition of vanillate. The cdbS-FLAG strain expresses this allele from the native site. The same amount of total protein was loaded per lane. PilC is used as a loading control. Numbers below show the mean ± STDEV of CdbS-FLAG normalized by the PilC level calculated from three independent experiments. *, P<0.05 in Student’s t test in which samples were compared to CdbS-FLAG expressed from the native site. All strains are parB+/parB-YFP merodiploid. B. Cell length and chromosome organization of strains of indicated genotypes. Cells were grown in 1% CTT broth in the presence and absence of vanillate as indicated. Cells grown in the presence of vanillate were analyzed 24hrs after addition of vanillate. Cell length measurements are included from three independent experiments indicated in different colored triangles and the mean based on all three experiments. Numbers above indicate cell length as mean ± STDEV from all three experiments. ns, not significant in 2way ANOVA multiple comparisons test. Total number of cells analyzed: 469–643. Lower panels, fluorescence microscopy images of cells stained with DAPI and synthesizing ParB-YFP. In the demographs, cells are sorted according to length, DAPI signals are shown according to the intensity scale, and ParB-YFP signals in pink. Scale bar, 5μm. N = 400 cells for all strains. All strains are parB+/parB-YFP merodiploid. (TIF) [file pgen.1010819.s003.tif]

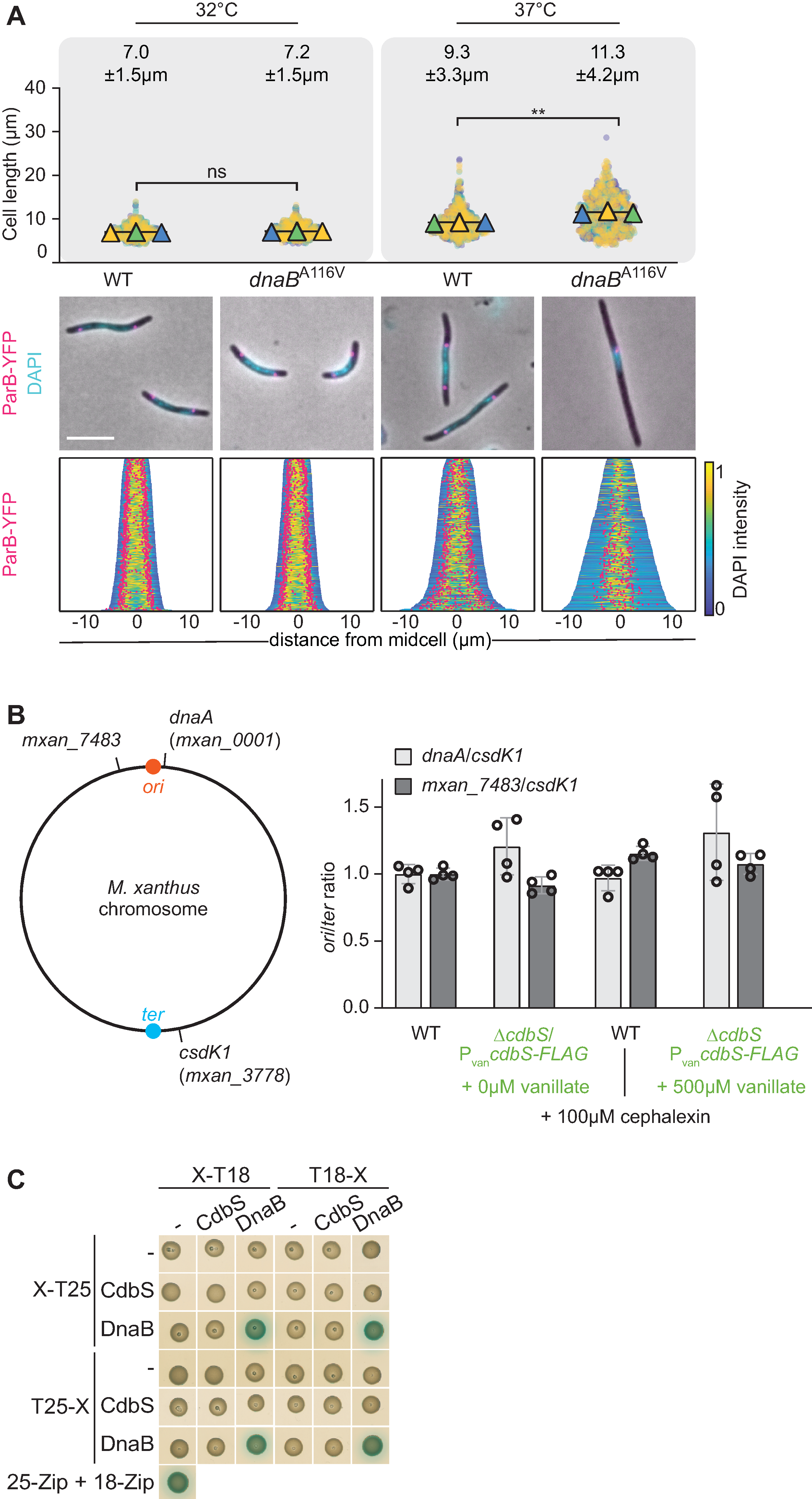

Supplement: S4 Fig — A. Cell length and chromosome organization of strains of indicated genotypes. Cells were grown at 37°C for 12hrs before the analysis. Cell length measurements are from three independent experiments indicated in different colored triangles and the mean is based on all three experiments. Numbers above indicate cell length as mean ± STDEV from all three experiments. ** P<0.0001, ns, not significant in 2way ANOVA multiple comparisons test. Total number of cells analyzed: 603–769. Lower diagrams, fluorescence microscopy images of cells stained with DAPI and synthesizing ParB-YFP. In the demographs, cells are sorted according to length, DAPI signals are shown according to the intensity scale, and ParB-YFP signals in pink. Scale bar, 5μm. N = 400 cells for all strains. All strains are parB+/parB-YFP merodiploid. B. qPCR analysis of ori/ter ratio in indicated strains. Left diagram, the positions on the M. xanthus chromosome of the primers used for determination of the ori/ter ratio. Right diagram, ratios are shown relative to the level in untreated WT as mean ± STDEV from four biological replicates with three technical replicates each. In pairwise comparisons, no significant differences were observed in 2way ANOVA multiple comparisons test. Both strains are parB+/parB-YFP merodiploid. C. BACTH analysis of CdbS and DnaB interaction. The indicated proteins were fused to the N-terminus and C-terminus of T25 or the N- and C-terminus of T18 as indicated. Blue and white colony colours indicate an interaction and no interaction, respectively. T25-Zip + T18-Zip, positive control; the strains in the row and column labelled “–”contain the indicated plasmid and an empty plasmid and served as controls for self-activation. The same results were observed in two biological replicates. (TIF) [file pgen.1010819.s004.tif]

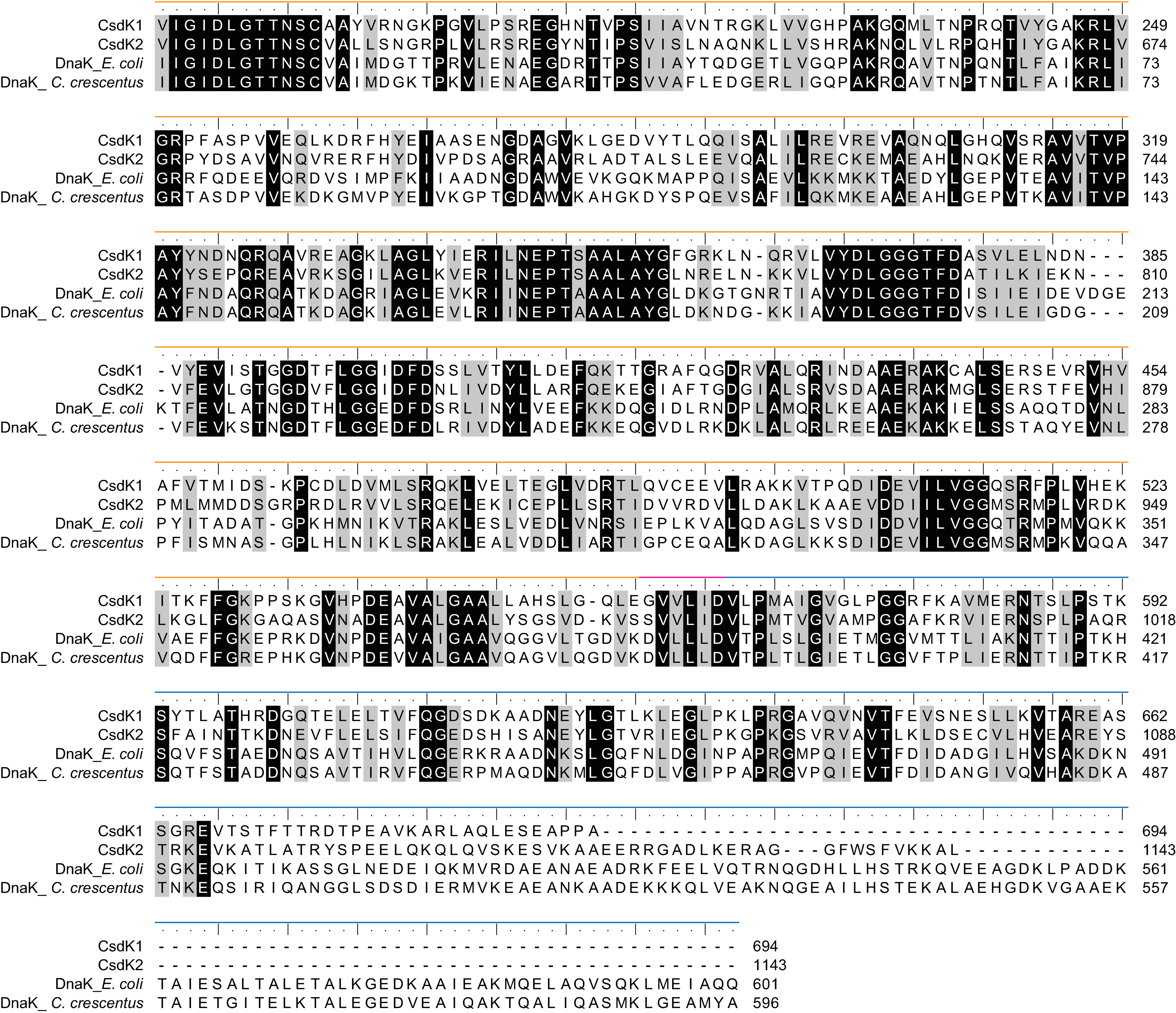

Supplement: S5 Fig — Alignment of the DnaK characteristic domains of CsdK1 and CsdK2 with those of DnaK proteins of E. coli and C. crescentus. The nucleotide-binding domain (orange), the linker (pink) and the substrate-binding domain (blue) are indicated. (TIF) [file pgen.1010819.s005.tif]

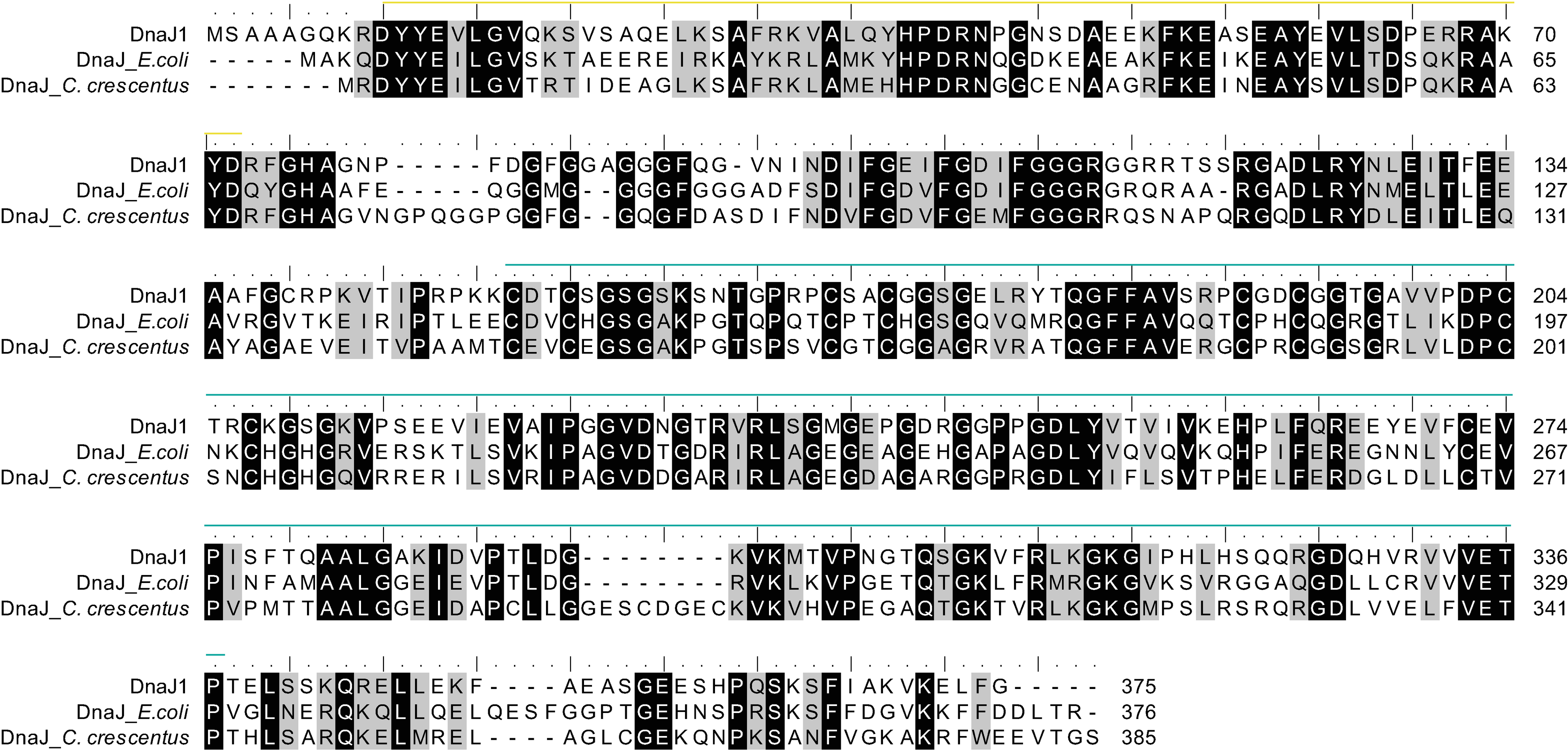

Supplement: S6 Fig — Alignment of full-length DnaJ1 with DnaJ of E. coli and C. crescentus. The J domain (yellow) and the J_central domain (turquoise) are indicated. (TIF) [file pgen.1010819.s006.tif]

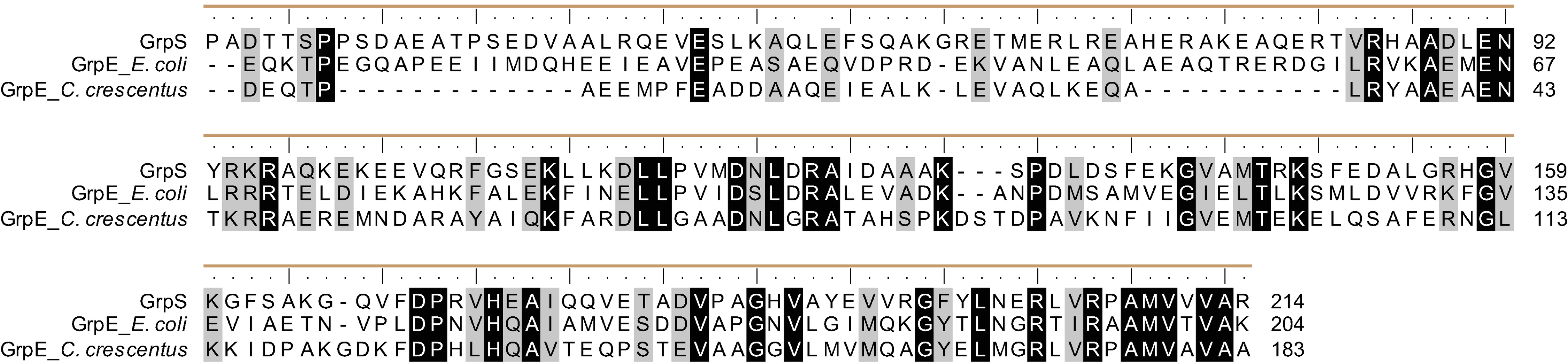

Supplement: S7 Fig — Alignment of the GrpE domain of GrpS with those of GrpE of E. coli and C. crescentus. The GrpE domain (brown) is indicated. (TIF) [file pgen.1010819.s007.tif]

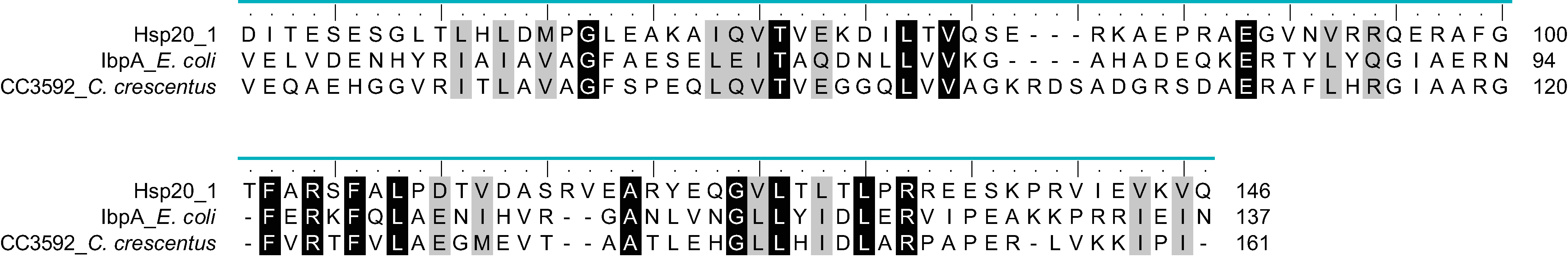

Supplement: S8 Fig — Alignment of the Hsp20 domain of Hsp20_1 with those of IbpA of E. coli and the domain of CC_3592 of C. crescentus. The Hsp20 domain (cyan) is indicated. (TIF) [file pgen.1010819.s008.tif]

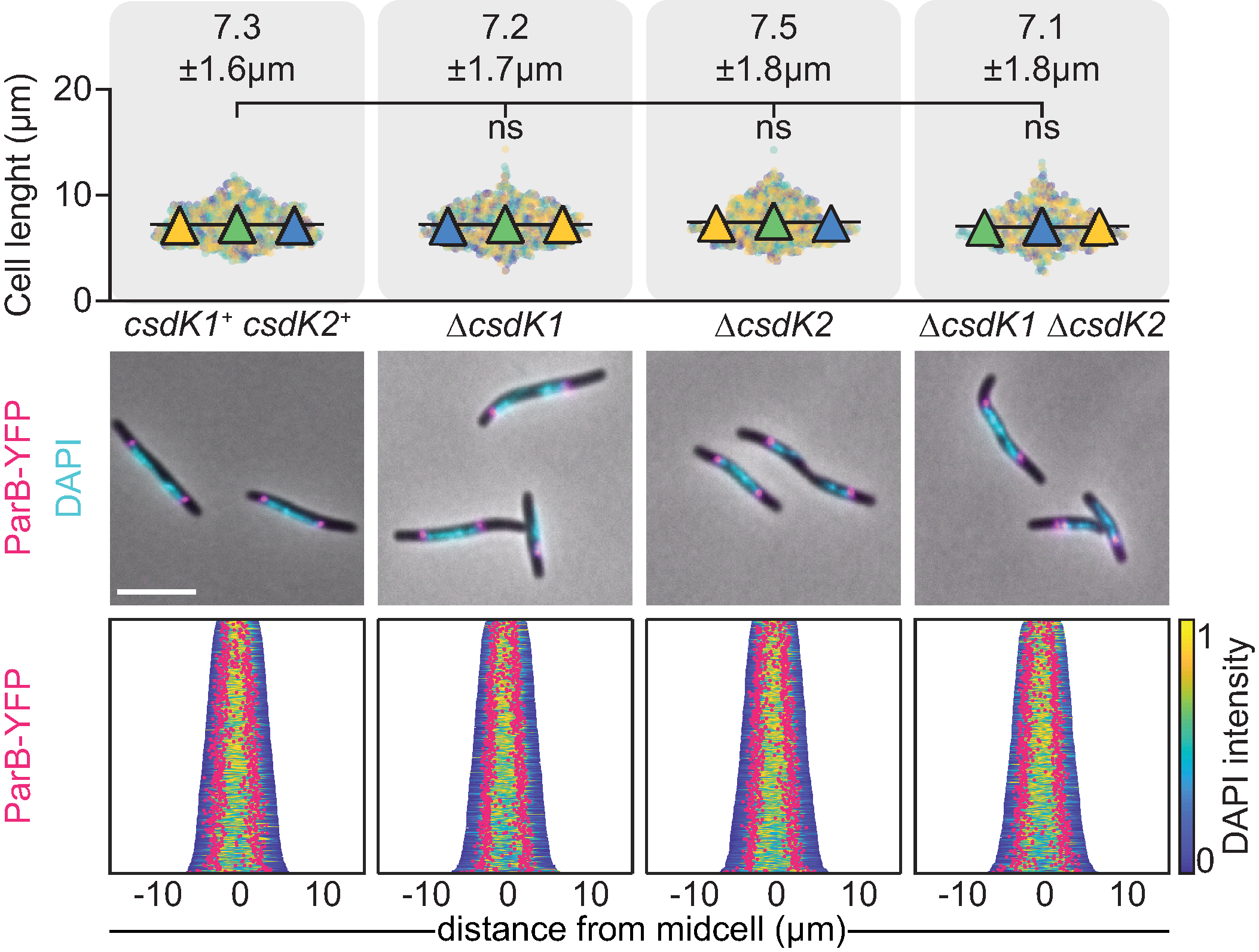

Supplement: S9 Fig — Cell length and chromosome organization of strains of indicated genotypes. Cell length measurements are included from three independent experiments indicated in different colored triangles and the mean calculated based on all three experiments. Numbers above indicate cell length as mean ± STDEV from all three experiments. ns, not significant in 2way ANOVA multiple comparisons test. Total number of cells analyzed: 540–719. Lower diagrams, fluorescence microscopy images of cells stained with DAPI and expressing ParB-YFP. In the demographs, cells are sorted according to length, DAPI signals are shown according to the intensity scale, and ParB-YFP signals in pink. Scale bar, 5μm. N = 400 cells for all strains. All strains are parB+/parB-YFP merodiploid. (TIF) [file pgen.1010819.s009.tif]

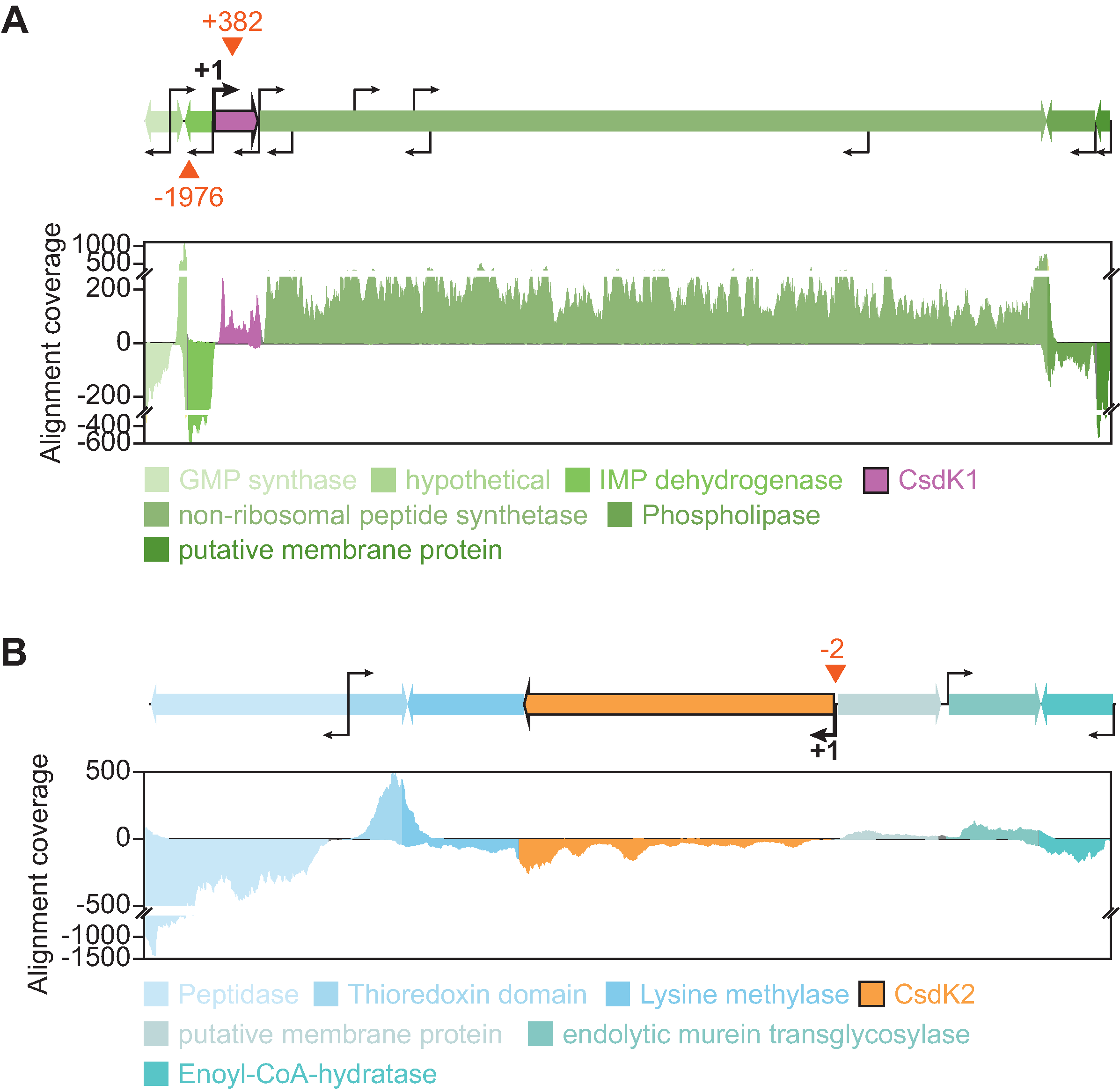

Supplement: S10 Fig — Organization of the csdK1 (A) and csdK2 (B) loci. A, B. Upper diagrams, transcription direction is indicated by the orientation of arrows, kinked arrows indicate transcription start sites as mapped in [24]. Coordinates indicate bp relative to the transcription start site of csdK1 and csdK2, respectively. The lower diagrams show data from RNAseq as base-by-base alignment coverage for total RNA isolated from cells growing in 1% CTT broth [24]. Positive and negative values indicate reads mapped to the forward and reverse strand, respectively. Reads assigned to a gene are colored according to the gene color code in the upper diagrams. Red triangles indicate the CdbA peak summits from a ChIP-seq analysis in which an active CdbA-FLAG protein was used as bait [11]. (TIF) [file pgen.1010819.s010.tif]

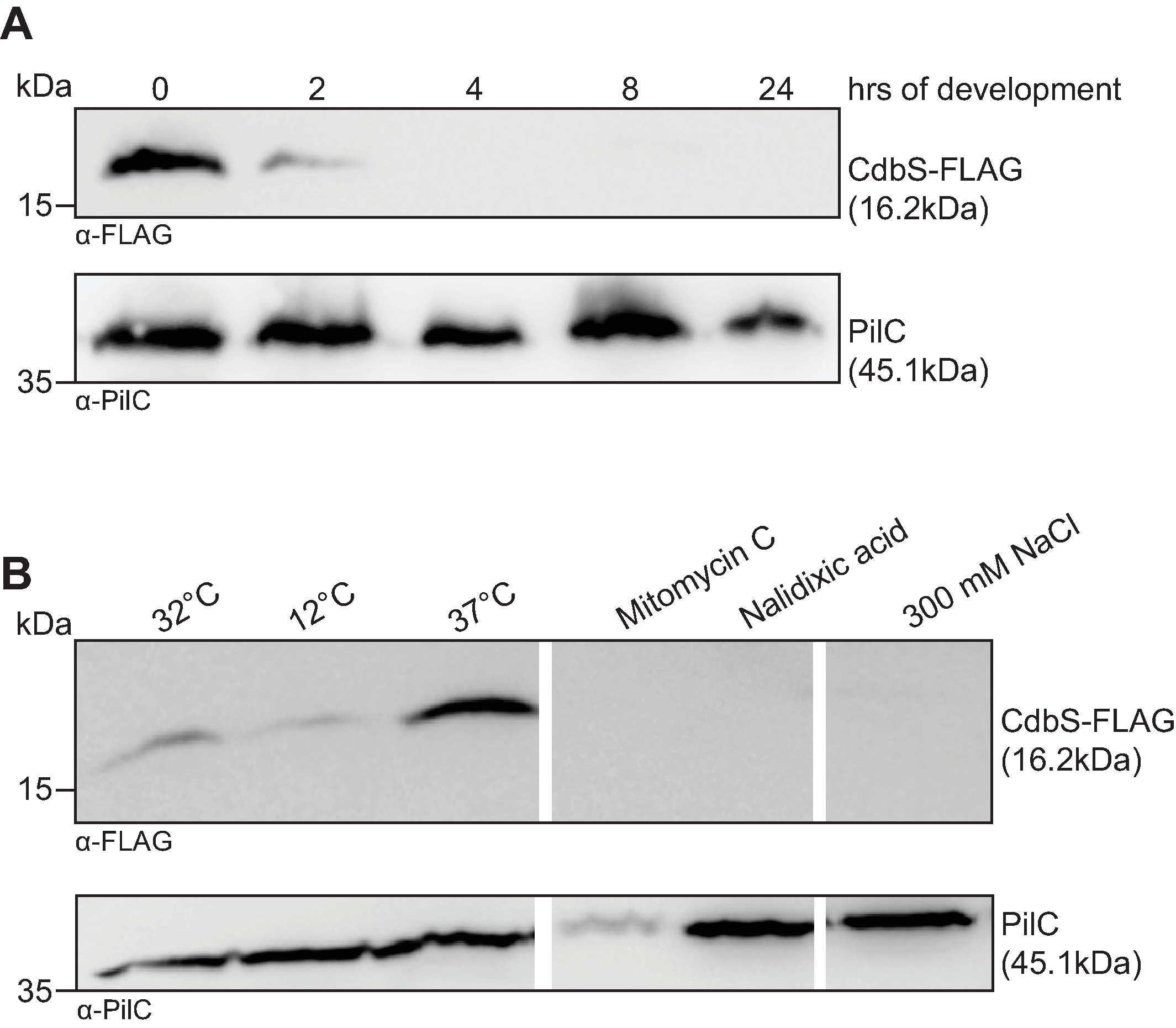

Supplement: S11 Fig — A. CdbS-FLAG accumulation decreases during development. Cells were developed under submerged conditions and harvested at the indicated time points. The same amount of protein was loaded per lane. PilC was used as a loading control. Similar results were obtained in two independent experiments. B. CdbS-FLAG accumulates at an increased level at 37°C. Cells were exposed to the indicated stresses for 18hrs and then harvested. The same amount of protein was loaded per lane. PilC was used as a loading control. Similar results were obtained in two biological replicates. CdbS-FLAG was synthesized from the native cdbS locus. All samples were loaded on the same gel; gaps indicate lanes removed for presentation purposes. (TIF) [file pgen.1010819.s011.tif]

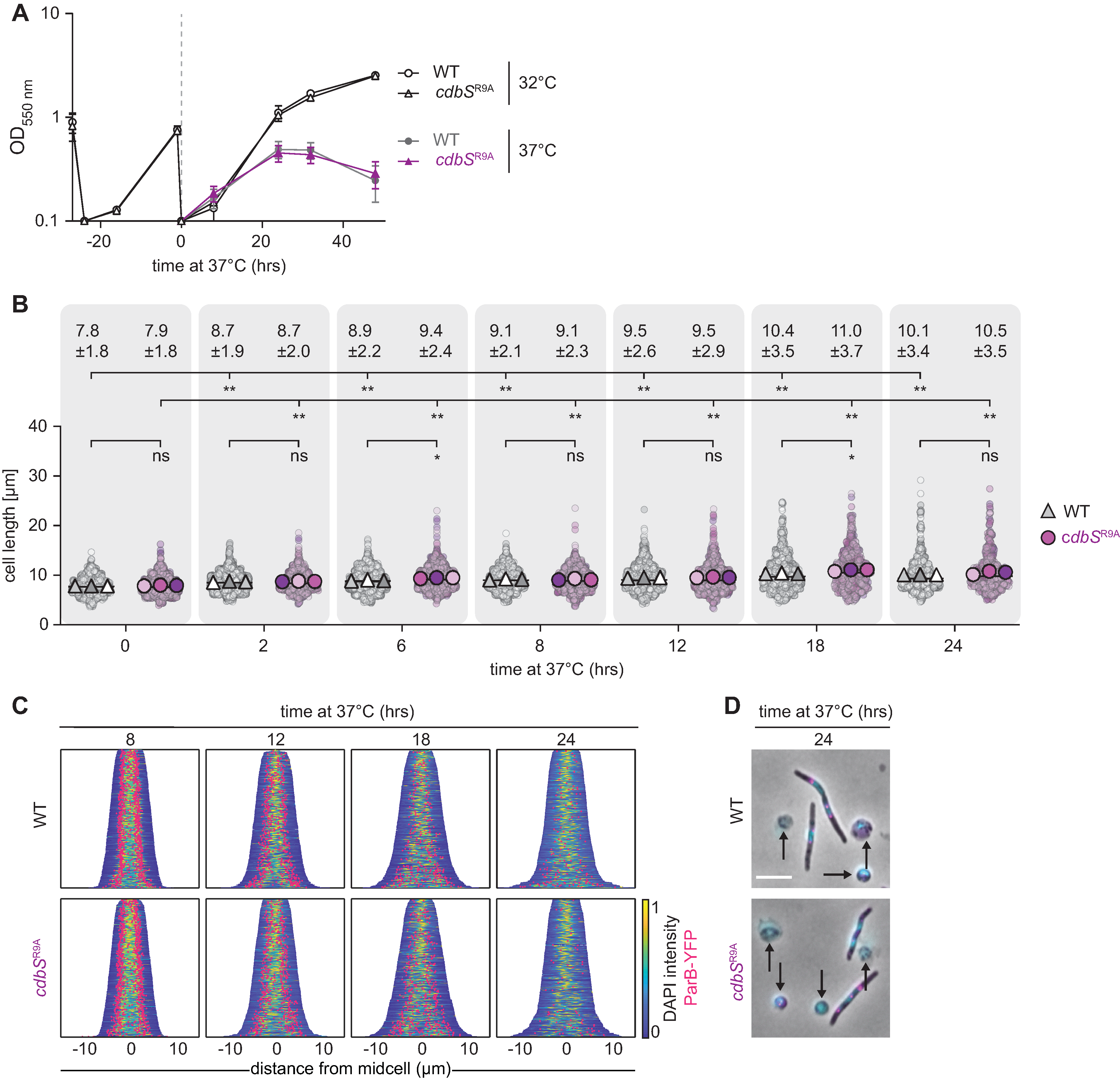

Supplement: S12 Fig — A. Growth of strains of indicated genotypes. Cells were grown in 1% CTT broth in suspension culture at the indicated temperatures. Growth curves were generated from three biological replicates. All strains are parB+/parB-YFP merodiploid. B, C. Cell length analyses (B) and chromosomes organization (C) in strains of the indicated genotypes during growth at 37°C. Cells were grown at 37°C for indicated periods. In B, cell length measurements are included from three independent experiments indicated in different colored triangles and the mean is based on all three experiments. Numbers above indicate cell length as mean ± STDEV calculated from all three experiments. *, P< 0.05, **, P< 0.01 and ns, not significant in 2way ANOVA multiple comparisons test. Only rod-shaped cells were included in the measurements. Total number of cells analyzed: 421–938. In C, only rod-shaped cells were included in the analysis and not cell that were undergoing lysis or had rounded up (See E) and cells are sorted according to length, DAPI signals are shown according to the intensity scale, and ParB-YFP signals in pink. N = 400 cells for all strains. D. Microscopic analysis of cells of the indicated genotypes at 37°C for the indicated period. Cells were stained with DAPI (blue signal) and synthesizing ParB-YFP (pink signal). Arrows point to cells that have lost their rod-shape and rounded up. Scale bar, 5 μm. A-D, both strains are parB+/parB-YFP merodiploid. (TIF) [file pgen.1010819.s012.tif]
