# Supplementary material for: During heat stress in Myxococcus xanthus, the CdbS PilZ domain protein, in concert with two PilZ-DnaK chaperones, perturbs chromosome organization and accelerates cell death
Source: PLoS Genet. 2023 Jun 20;19(6):e1010819. doi: 10.1371/journal.pgen.1010819 (PMC10313047; doi:10.1371/journal.pgen.1010819)
Supplement: S1 Table — (DOCX) [file pgen.1010819.s014.docx]

**S1 Table.** Primers used in this study.

| **Primer name** | **Sequence (5’→3’)** |
| --- | --- |
| CdbS_E | GAGGCGCTCCAGTACATCGC |
| CdbS_F | CCGCCAACATCCTGTCGCAG |
| CdbS_G | ACCTCCGTTTCGACAAGGTC |
| CdbS_H | GTCGTCCTGCGTCTCATAGG |
| CdbA_E | GAAGCCGGAGTCGCTC |
| CdbA_F | GCCAGCCGAAAGGCTC |
| CdbA_G | CAGTCGCTGTACTTCCCC |
| CdbA_H | CTCGTCGCCAGTCACA |
| CsdK1_E | CACCTTCACCGCGTCCACGC |
| CsdK1_F | GGGCTCCGCGCCGCAGAA |
| CsdK1_G | TTCCCCCGGTTCCTCCCAAT |
| CsdK1_H | ATGGAGGGCACCGTGTTGTG |
| CsdK2_E | TGTGAACCACGCCAGGCTGCC |
| CsdK2_F | GCAGTGCCCGTCCTCGCGCTT |
| CsdK2_G | AAGGGCTTCCCCAAGTGCGCC |
| CsdK2_H | TTGAGCTCGCGGTTGAGACCG |
| 4328 fw_NdeI | ATGCATATGATGTTTGAGCGTCCTCACGA |
| oMS002 | ATGTCTAGAATGTTTGAGCGTCCTCACGAGCGCGCGTCCCACCTC |
| oMS004 | GTTAAGCTTTCAGTGCATCACCCGCTCGCGGTCG |
| oMS013 | ctgcccgccgcgctgcccgcgctgccgtgcatcacccgctcgcggtc |
| oMS024 | gataagcttggttcagcccggaatcgtgcc |
| oMS025 | gcgggcagcgcggcgggcagcggcgagttcGACTACAAGGACGACGACGACAAGtgacgtccgcccggggcccgtgagg |
| oMS026 | cagtgaattcgagctcatcccggacatc |
| oMS029 | gcgggcagcgcggcgggcagcggcgagttcatgagcaaaggagaagaactt |
| oMS041 | GAGGAATTCTCACTTGTCGTCGTCGTCCTTG |
| oMS068 | ccttctagagaggcgtgtcatcgtcacccg |
| oMS068 | CCTTCTAGAGAGGCGTGTCATCGTCACCCG |
| oMS069 | gcctgcatgctcacttgtacagctcgtcca |
| oMS070 | tacgaattcgagctcggtaccggggatcctctagagtcgacctgcaggcatgcaagcttggcac |
| oMS071 | gtgccaagcttgcatgcctgcaggtcgactctagaggatccccggtaccgagctcgaattcgta |
| oMS082 | GCCAAGCTTCGGACCGCGGTGGAATGGAG |
| oMS083 | TCTTCTAGAGGATCCCTCCTCCCGAGGGTCCTGGC |
| oMS084 | GAGGGATCCTCTAGAAGATCTGCTAGCGGCAGCGCGGCGGGCAGCGGCATGGTGAGCAAGGGCGAGGAG |
| oMS085 | GTCCGTCGTAGCCATCCGTCGTTACTTGTACAGCTCGTCCATG |
| oMS086 | CATGGACGAGCTGTACAAGTAACGACGGATGGCTACGACGGAC |
| oMS087 | AGTGAATTCGAGACGGCTATGGCACGGCGC |
| oMS092 | gccaagcttatcggttatacggacatcaag |
| oMS093 | cttcaccttggcgatgtcgcgtttctgacccgccgc |
| oMS094 | atcgccaaggtgaaggagctg |
| oMS095 | agtgaattcgaagaagaggacgccggcctg |
| oMS096 | caaccctgcggacaggttcag |
| oMS097 | ggcgtcatccgcccgcgaatg |
| oMS098 | gaaatctgtctccgcgcagga |
| oMS099 | ctccgcgaacttctccagcag |
| oMS100 | gccaagcttgagcgcctcctcaaggaagtg |
| oMS101 | ggaagagttctcggacttgtcgttcgagccggccac |
| oMS102 | gtccgagaactcttccggggggagtcag |
| oMS103 | cagtgaattcgcaggacactgagcccggcga |
| oMS104 | gatgactgccccatggaggag |
| oMS105 | agctccaggatggagatatcg |
| oMS106 | cattgacgcggcggttcgcag |
| oMS107 | ctcggtgtcggtcgtggcggtg |
| oMS116 | gccaagcttatgatgcgcgacagacgtccg |
| oMS117 | gacttcgatgacgcgggcggagttgaacggattgcgagtctgcat |
| oMS118 | cgcgtcatcgaagtcaaggtc |
| oMS119 | agtgaattccctgcgcacgttcctcatcac |
| oMS120 | gcaccagcgtcttgccgttgtc |
| oMS121 | cagcggaatcatgcagcaagag |
| oMS122 | cgagctgacgcagcccatgtg |
| oMS123 | gtcagcgtcagcacaccctgc |
| oMS131 | GACTCTAGAGATGTTTGAGCGTCCTCACGAG |
| oMS132 | GCTCGGTACCTTGTGCATCACCCGCTCGCGGTC |
| oMS133 | GACTCTAGAATTGACGGAATCGAATCAGGCG |
| oMS134 | CTCGGTACCTTGGCTCTTCCGAACAACCCCTTG |
| oMS135 | GACTCTAGAAGTGGTCTGCCCACCTGTCGAC |
| oMS136 | ATTGAATTCTTCTTCCTGCCCAGCGCCTTCTT |
| oMS141 | ATTGAATTCTTAGTTCTTCCTGCCCAGCGCCTTCTT |
| oMS154 | GACTCTAGAGATGGAGAACGTCCACGAG |
| oMS155 | CTCGGTACCAGCTCGCTCCGCGACCGGCTC |
| oMS177 | gccaagcttgacgaagagctacaccctg |
| oMS180 | cagtgaattccgcgagtgagaggatgc |
| oMS182 | gccaagcttgtcaacgcggacgaagcc |
| oMS183 | tgggacgtcgtatgggtacttcctgcccagcgcct |
| oMS184 | tacccatacgacgtcccagactacgcttgatggtcacatcctcaattcgaaaag |
| oMS185 | Cagtgaattccgctggagcaccgacaac |
| oMS187 | gataagcttgtggtgcccatcgcgtc |
| oMS188 | ccgcgctgcccgcgctgccctcgctccgcgaccggctc |
| oMS189 | CAGCCCTCCGTGTTCGCG |
| oMS190 | gtagaattcccaccaccagtcggtgc |
| oMS191 | gcatcggcaagacgtcc |
| oMS192 | cggtgaagtgcggcatg |
| oMS193 | gaacacggagggctgttatttgtagagctcatc |
| oMS194 | cgggcagcgcggcgggcagcggcgagttcatggtgagcaagggcgag |
| oMS204 | gatcaaggcagggccaccacggacagg |
| oMS205 | Cctgtccgtggtggccctgccttgatc |
| oMS206 | Gtagaattcgggctcacgtcctcggcca |
| oMS207 | gtatctagaagactacaaggacgacgacgacaagctggacatcgatgagcc |
| oMS208 | gtagaattcctatgggctcacgtcctcggccag |
| oMS211 | CTGTTCGCATATGTCACTTGTACAGCTCGTCCA |
| oMS212 | AAGTGACATATGCGAACAGAAAGTAATCGTATGCTCTGCGGGGTGAAGAC |
| oMS228 | gaaaacgcgcactgcgtg |
| oMS231 | ccgcgctgcccgcgctgccggctcttccgaacaaccc |
| oMS232 | cgcagtgcgcgttttcacttgtacagctcgtccatg |
| cdbS qPCR fwd | CCGTTTCGACAAGGTCTTCA |
| cdbS qPCR rev | GGACCTCCACGAACATGC |
| csdK1_ter 1 qPCR fwd | TTCCACTATGAGATCGCGGC |
| csdK1_ter 1 qPCR rev | CTGATGGCCCAACTGGTTCT |
| csdK2 qPCR fwd | GGGGATCGAGGGGTCAAAAC |
| csdK2 qPCR rev | GCTTCAGCTTCA |
| rpsS qPCR fwd | GTTCGATCAAGAAGGGTCCGT |
| rpsS qPCR rev | GACGAACACCGGGATGAACT |
| ori 1/dnaA fwd | AACCTCATCTGGGAGCGAGA |
| ori 1/dnaA rev | TTGCCGAGGAACTGGATGTC |
| ori 2/7483 fwd | TGCCCACCATCAATCCATCC |
| ori 2/7483 rev | TGAGTTCCTGACGCTTGGTG |
