# Supplementary material for: During heat stress in Myxococcus xanthus, the CdbS PilZ domain protein, in concert with two PilZ-DnaK chaperones, perturbs chromosome organization and accelerates cell death
Source: PLoS Genet. 2023 Jun 20;19(6):e1010819. doi: 10.1371/journal.pgen.1010819 (PMC10313047; doi:10.1371/journal.pgen.1010819)
Supplement: Attachment — Submitted filename: Seidel_Rebuttal.docx [file pgen.1010819.s016.docx]

**Reviewer #1:** The manuscript by Seidel et al., describes a follow up study of the work of Skotnicka et al, published in Nature Communications in 2020. Previously, the authors identified CdbA as an essential c-di-GMP binding protein and address here the question why CdbA is essential in Myxococcus xanthus. Using an accurate suppressor screen, they identify CdbS - a protein of previously undefined function - which mediates cell toxicity upon CdbA deletion. They confirm using a set of mutants and overexpression strains that CdbS accumulates upon CdbA depletion and causes defects in chromosome organization and consequently cell death. Moreover, they provide detailed mechanistic understanding of how and why CdbS accumulates when CdbA levels decrease. Using a pull-down assay they identify two chaperons, CsdK1 and CsdK2, the expression of which is increased in the cdbA depletion strain and which interact with CdbS and stabilize the protein. Moreover, the authors show that heat stress (growth at 37°C) induces the CdbS/CsdK1+2 system and contributes to cell death.

I really enjoyed reading this manuscript. The experiments are very well performed, important controls are presented and the manuscript is very well written. The work provides many mechanistic insights and covers a very interesting field on NAPs and PilZ-domain containing proteins. Many bacteria e.g. Vibrio cholerae have multiple PilZ domains, but our knowledge about the physiological role of these domains is still very limited.

The only aspect which I personally found a bit “disappointing” is that there are no hints about what could be the function of CdbS and how this protein might affects chromosome organization. Obviously, the authors are aware about this limitation and discuss this point and I understand that this question can only be addressed in a follow-up study.
**Response:** Thank you so very much for the very positive & constructive feedback. In the revised manuscript, we amended the Discussion of the possible function of the CdbA/CsdK1/CsdK2/CdbS system (line 552-575). In line 593-609, we describe our ideas about how CdbS could possibly affect chromosome organization. We did not change this discussion because we clearly describe a way forward to address this question.

There are only few minor comments that I would like to make:
1. The authors often use the term “disrupted chromosome organization” and I do not fully understand what exactly is meant here. In line 259, they say “nucleoid as well as ParB-YFP cluster localization were highly disorganized” and refer to Fig. 3DE. I find it very difficult to see what the authors mean in the images provided. Maybe some zoom-in images can be provided to make this point more clearly?
**Response:** We apologize for not being clearer. Throughout the revised text, we have included more careful descriptions of the “chromosome phenotypes”. Also, we included new images for the strains in Fig. 3E, lower panels to more clearly visualize the defects in chromosome organization.

2. The cascade described here covers many different players so I was wondering whether the authors can provide something like a summary image that covers the relevant components and signals. This would certainly be helpful for better understanding of the reader.
**Response:** This is a great suggestion. Thank you. We included a new Fig. 7 in which we summarize the three states of the CdbA/CsdK1/CsdK2/CdbS system.

3. Line 156: sentence that starts with “By immunoblot…”- a verb is missing.
**Response:** We have been looking at this sentence, and we think the verb is there (now line 158-159).

**Reviewer #2:** This manuscript by Seidel et. al. explores the molecular mechanism of essentiality of a cyclic di-GMP (c-di-GMP) binding nuclear-associated protein CdbA. To understand why this protein is essential, the authors performed suppressor analysis and found that lethality is due to a new c-di-GMP binding protein, CdbS. The authors find that CdbS accumulates when CdbA is depleted due to the chaperones CsdK1/CsdK2. The authors uncover many new molecular pathways, although strangely they do not seem connected to c-di-GMP, even though all of these proteins bind to c-di-GMP (with the exception of CsdK1). They also importantly show that this pathway is activated during growth at 37 deg C, although they do not give any plausible explanation for how this could be adaptive (See point 5). The data are impressive and solid and support the author’s conclusions. They answer many questions about this system, but significant questions remain such as how CdbS causes cell death and the role of c-di-GMP. However, I appreciate they cannot figure out all aspects of this complex system in one manuscript. But I do think testing their proposed model for increased c-di-GMP triggering this system at 37 deg C would be within the scope of this manuscript (point 4). In sum, this is an interesting, well done paper that should be impactful to the microbiology community.
**Response:** Thank you so very much for the very positive & constructive feedback.
In the revised manuscript, we have amended the Discussion of the possible function of the CdbA/CsdK1/CsdK2/CdbS system (line 552-575).

**1.** Fig. S4B would be more convincing with a positive control that alters the ori/ter ratio. A thymine auxotroph mutant that becomes starved for thymine would suffice.
**Response:** Thank you for this suggestion. The reviewer’s question is important and addresses the overall question of how an elevated CdbS level perturbs chromosome organization, thereby, eventually causing cell death.

The experiments included in the manuscript (1) demonstrate that the *dnaB*^A116V^ mutant at the non-permissive temperature has a highly condensed nucleoid morphology that is very different from that of cells with an elevated CdbS level (Fig. S4A); (2) support that a strain with an elevated CdbS level replicates as WT and that an elevated CdbS level neither inhibits nor stimulates DnaB function (Fig. S4B); and, (3) support that CdbS and DnaB do not interact based on a BACTH assay (Fig. S4C). Therefore, we conclude “how CdbS and DnaB might be connected remains unclear” (line 329-330).

Unfortunately, we do not have a *M. xanthus* thymine auxotroph. Thus, doing the suggested experiment would require generating such a mutant and characterizing it in detail before being able to do the suggested experiment. Based on the experiments already included in the manuscript, we believe that further investigating a potential DnaB/CdbS interaction might best be performed in future experiments focusing specifically on how an elevated CdbS level interferes with chromosome organization. We would like to add that we conclude (line 329-330) “how CdbS and DnaB might be connected remains unclear” and in the Discussion we write (line 596-597) “our results do not provide support for a direct interaction between CdbS and DnaB”. Thus, we do not rule out that the two proteins could interact.

2. Line 398-400-I think it reasonable to propose that CsdK1 and CsdK2 stabilize CdbS, but I don’t understand the argument that because accumulation of CdbS occurs post-transcriptionally, this rules out that these proteins do not impact translation.
**Response:** Thanks for pointing this out to us. We rewrote the text (line 393) to “CsdK1 and CsdK2 enable the increased CdbS accumulation during CdbA depletion”. Moreover, we rewrote the argument in which we suggest that CsdK1 and CsdK2 stabilize CdbS (line 410-413) “Because this increased CdbS accumulation is post-transcriptionally regulated, and CsdK2 interact with CdbS in the BACTH analysis, we suggest that these two DnaK chaperones stabilize CdbS during CdbA depletion rather than increasing the translation of the *cdbS* mRNA”. Finally, we return to this point in the Discussion (line 617-619) and in the new Fig 7. In all case, we suggest that CsdK1 and CsdK2 stabilize CdbS, i.e. we do not state that we have demonstrated this.

3. One experiment that is missing that would significantly support their model is to overexpress CdbS in the CsdK1/CsdK2 cdA-mCh strain. If these protein function solely through enhancing CdbS levels, then overexpression of this protein should restore sensitivity to vanillate depletion.
**Response:** Thank you for this suggestion. Our take of the experiment suggested by the reviewer is to deplete cells of CdbA-mCh in the absence of CsdK1 and CsdK2 and at the same time overexpress CdbS-FLAG to test whether these cells are still sensitive to CdbA-mCh depletion. In *M. xanthus*, the best-characterized and most tightly regulated promoter is P_van_. Therefore, we use this promoter both for depletion of CdbA-mCh and for overexpression of CdbS-FLAG. Consequently, with these two constructs, we cannot deplete CdbA-mCh and overexpress CdbS-FLAG in the same cells. So, for purely technical reasons we would not be able to do the suggested experiment. In addition to P_van_, there are also somewhat characterized IPTG-regulated and Cu-regulated promoters in *M. xanthus*. However, we do not know whether these promoters are sufficiently strong and tight to do the proposed experiment. Thus, doing the proposed experiment would require generating several new plasmids and strains and characterizing them in detail before being able to do the actual experiment. Also, based on the experiments in Fig. 5B-C (in which we deplete cells of CdbA-mCh (using P_van_) ±CsdK1 and ±CsdK2 and find that in the absence of both CsdK1 and CsdK2, CdbS-FLAG only increases ~1.6-fold) it is not clear to us that CdbS-FLAG would be able to accumulate at an increased level in the suggested experiment.

Finally, we would like to add that (1) the Δ*cdbS* mutation suppresses the CdbA-mCh depletion phenotype (Fig. 1A-B); (2) the Δ*csdK1* Δ*csdK2* mutations jointly suppress the CdbA-mCh depletion phenotype (Fig. 4D & 5B-C), and (3) CdbS-FLAG over-accumulation is sufficient to disrupt chromosome organization and cause cell death (Fig. 3C-E). Based on the experiments already included in the manuscript, we believe that further investigating whether CsdK1/CsdK2 have functions other than stabilizing CdbS during CdbA-mCh depletion might best be performed in future experiments focusing specifically on how an elevated CdbS level interferes with chromosome organization and eventually cause cell death.

4. Lines 463-470-The authors propose a model whereby high temperatures induce the activity of CdbS not by a decrease in CdbA but by an increase in c-di-GMP concentrations. But the authors do not test this model, which seems straightforward and would be a nice cap to the manuscript. One way they could test this model is by increasing temperature in the c-di-GMP binding mutant of CdbA, which should not have the death phenotype at higher temperatures.
**Response:** Thank you for this suggestion. We have previously shown that DNA and c-di-GMP binding by CdbA involve the same interface of the tetramer and is mutually exclusive *in vitro*. Therefore, CdbA variants that cannot bind c-di-GMP do not bind DNA *in vitro* and, consequently, they are completely non-functional *in vivo.* In other words, strains that only express a non-c-di-GMP-binding CdbA variant are not viable, i.e. they phenocopy the strain depleted of CdbA. Therefore, we cannot do the suggested experiments because the cells containing a non-c-di-GMP-binding CdbA variant are dead even before we raise the temperature.

To make it more clear that CdbA variants that cannot bind c-di-GMP also do not bind DNA and are non-functional *in vivo*, we rewrote (line 118-122) “DNA and c-di-GMP binding by CdbA involve the same interface of the tetramer and is mutually exclusive *in vitro* [11]. Consistently, CdbA variants that cannot bind c-di-GMP do not bind DNA *in vitro* and, consequently, these variants are non-functional *in vivo* and do not support proper chromosome organization [11]”.

5. Lines 540-547-I appreciate the authors do not understand why this systems promotes death at 37 deg Celsius, but they do not offer any reasonable explanation or speculation. Death would only be favored if it contributes to fitness of related neighbors. Why would this be the case at 37 deg C? Perhaps this phenotype is media specific and only observed in the lab in rich growth media whereas more physiologically relevant environments for Myxo would reveal the fitness benefit of activation of this pathway.
**Response:** Thank you for gently pushing us to think harder about the function of the CdbA/CsdK1/CsdK2/CdbS system. In the revised manuscript, we have amended the Discussion of the possible function of the CdbA/CsdK1/CsdK2/CdbS system (line 552-575).

**Reviewer #3:** The manuscript by Seidel et al. identified a PilZ-like protein, CdbS, which when stabilized by two chaperones, CsdK1 and CsdK2, accelerate cell death under heat stress. This work revealed why CdbA, a nucleoid-associated protein, is essential for M. xanthus. Overall, this manuscript contains substantial data which are logically organized and presented. I have only a few minor comments.
**Response:** Thank you so very much for the very positive & constructive feedback.

1. The title. Exactly how the over-accumulated CdbS triggers cell death is unknown. I do agree with the authors that the phenotype of CdbS over-accumulation is related to the mis-organization of chromosome. However, there is no evidence that CdbS, or CsdK1 and CsdK2, directly perturb chromosome. Thus, the title is misleading.
**Response:** Thank you for bringing up this point. Because we do not say that CdbS, in concert with CsdK1 and CsdK2, *directly* perturb chromosome organization and accelerates cell death, we believe that the title is still correct.

2. It is hard to believe that although CdbS and CsdK2 bind c-di-GMP, their functions are not affected by c-di-GMP at all. The role of c-di-GMP needs to be (at least) better discussed. What is the estimated copy number of CdbS? Rather than being an unlikely "toxin/antitoxin" partner for CdbA, could CdbS function as a sink for c-di-GMP, which promotes CdbA to bind DNA and thus slows down the over-accumulation of CdbS?
**Response:** Concerning the idea that the CdbA/CsdK1/CsdK2/CdbS system could be a toxin/antitoxin system, we apologize for not being clearer. In the revised manuscript, we have amended the Discussion of the possible function of the CdbA/CsdK1/CsdK2/CdbS system (line 552-575) including why we find it unlikely that it is a toxin/antitoxin system.

Concerning the idea that CdbS would be a sink for c-di-GMP, we believe that this is unlikely because cells expressing the non-c-di-GMP binding variant of CdbS in all *in vivo* experiments are indistinguishable from cells expressing the WT CdbS protein.

Concerning the copy number of CdbS: We have not determined this number. We believe that at this point, this is not essential information. Also, we believe that this question is better addressed in future analyzes to address how an increased level of CdbS causes defects in chromosome organization and eventually cell death.

Concerning the role of c-di-GMP: In the revised manuscript, we have amended the Discussion of the possible function of c-di-GMP (line 627-648).

3. Some small grammar issues. Line 587, the increased accumulation of CdbS. Line 593: c-di-GMP binding by the ...
**Response:** Many thanks & done (line 617 and 624).
